# Plastid ribosomal protein S5 is involved in photosynthesis, plant development, and cold stress tolerance in Arabidopsis

**DOI:** 10.1093/jxb/erw106

**Published:** 2016-03-22

**Authors:** Junxiang Zhang, Hui Yuan, Yong Yang, Tara Fish, Sangbom M. Lyi, Theodore W Thannhauser, Lugang Zhang, Li Li

**Affiliations:** ^1^College of Horticulture, State Key Laboratory of Crop Stress Biology for Arid Area, Northwest A&F University, Yangling, 712100, China; ^2^Plant Breeding and Genetics Section, School of Integrative Plant Science, Cornell University, Ithaca, NY 14853, USA; ^3^Robert W. Holley Center for Agriculture and Health, USDA-ARS, Cornell University, Ithaca, NY 14853, USA

**Keywords:** Arabidopsis, cold stress tolerance, photosynthesis, plastid ribosomal protein, proteomics, RPS5.

## Abstract

Plastid ribosomal proteins are essential components of protein synthesis machinery and have diverse roles in plant growth and development. Mutations in plastid ribosomal proteins lead to a range of developmental phenotypes in plants. However, how they regulate these processes is not fully understood, and the functions of some individual plastid ribosomal proteins remain unknown. To identify genes responsible for chloroplast development, we isolated and characterized a mutant that exhibited pale yellow inner leaves with a reduced growth rate in Arabidopsis. The mutant (*rps5*) contained a missense mutation of plastid ribosomal protein S5 (RPS5), which caused a dramatically reduced abundance of chloroplast 16S rRNA and seriously impaired 16S rRNA processing to affect ribosome function and plastid translation. Comparative proteomic analysis revealed that the *rps5* mutation suppressed the expression of a large number of core components involved in photosystems I and II as well as many plastid ribosomal proteins. Unexpectedly, a number of proteins associated with cold stress responses were greatly decreased in *rps5*, and overexpression of the plastid *RPS5* improved plant cold stress tolerance. Our results indicate that RPS5 is an important constituent of the plastid 30S subunit and affects proteins involved in photosynthesis and cold stress responses to mediate plant growth and development.

## Highlight

Plastid RPS5 affects proteins involved in photosynthesis and translation machinery and mediates cold stress tolerance in Arabidopsis.

## Introduction

Ribosomes are large ribonucleoprotein complexes that are essential for protein synthesis in all living cells. Cytoplasm, plastids, and mitochondria are the three major locations for protein synthesis in plants. Plastid protein synthesis utilizes a bacterial-type 70S ribosome that comprises one small (30S) and one large (50S) ribosomal subunit (Schippers and Mueller-Roeber, 2010; Tiller and Bock, 2014). The chloroplast 30S subunit contains a single 16S rRNA and 24 plastid ribosomal proteins (RPs). The 50S subunit consists of three rRNAs (23S, 5S, and 4.5S) and 33 plastid RPs. While all four chloroplast rRNAs are encoded by the plastid genome, the plastid RPs are encoded by both plastid and nuclear genomes. Of the 24 RPs in the 30S small subunit (RPS), half are nuclear encoded and the remainder are encoded by the plastid genome (Yamaguchi *et al.*, 2000). However, 24 of the plastid RPs in the 50S large subunit (RPL) are encoded by the nuclear genome, whereas only nine are encoded by the plastid genome (Yamaguchi and Subramanian, 2000). Plastid RPs have been shown to play versatile roles in plant growth and development (Fleischmann *et al.*, 2011; Tiller *et al.*, 2012). While some RPs are not essential for ribosome accumulation and translation, the others are required for basal ribosome activity and influence specific processes in plant growth and development (Tiller and Bock, 2014).

A range of phenotypic effects are observed as a consequence of the lack of some individual plastid RPs, as shown with cytosolic RPs (Carroll, 2013; Schippers and Mueller-Roeber, 2010). Seven Arabidopsis plastid RP mutants (*prps20* and *prpl1*, *l4*, *l21*, *l27*, *l28,* and *l35*) are embryo lethal, indicating an essential role of these plastid proteins in embryo development (Romani *et al.*, 2012; Yin *et al.*, 2012). The tobacco *prps18* knockout mutant shows misshapen leaves and abnormal leaf blades, which reveals the importance of this plastid protein in leaf development (Rogalski *et al.*, 2006). The *rps21* mutation in Arabidopsis impairs photosynthesis and chloroplast development, and is hypersensitive to glucose (Morita-Yamamuro *et al.*, 2004). A defect in photosynthesis, pale green leaves, and a drastic reduction in growth rate are observed in the Arabidopsis *prpl11* mutant (Pesaresi *et al.*, 2001) and a number of other *prp* mutants (*prps1*, *prps17*, and *prpl24*) (Romani *et al.*, 2012; Tiller *et al.*, 2012). While some plastid RP gene mutations cause reductions in rRNA levels with impaired ribosome function, some others do not affect plastid rRNA accumulation and the assembly of plastid polysomes (Pesaresi *et al.*, 2001; Romani *et al.*, 2012; Tiller *et al.*, 2012). Clearly, these studies reveal diverse roles of plastid RPs in plant growth development. However, how plastid RPs regulate these processes is not fully understood, and the functions of some individual plastid RPs remain largely unknown.

Chloroplasts, as the most conspicuous plastid type, carry out many essential metabolic processes, including photosynthesis, fatty acid synthesis, amino acid synthesis, and carotenoid metabolism, and play an important role in plant development (Rolland *et al.*, 2012). To identify genes that affect chloroplast development and pigment synthesis, in this study we screened an Arabidopsis ethyl methanesulfonate (EMS)-mutagenized M_2_ population for leaf color mutants. Here, we report the isolation and functional characterization of a pale yellow inner leaf *rps5* mutant in Arabidopsis, which encoded a plastid RP S5 (RPS5). *rps5* showed serious impairment of chloroplast 16S rRNA processing and accumulation. A global and quantitative examination of proteomes via comparative proteomic analysis revealed that RPS5 affected the accumulation of a large number of photosystem I and II proteins and plastid RPs. In addition, it affected some proteins associated with cold stress responses, and overexpression of *RPS5* resulted in enhanced cold tolerance. Our data reveal that RPS5 is important for plastid ribosome function and is involved in photosynthesis, plant development, and cold stress resistance in Arabidopsis.

## Materials and methods

### Plant materials and growth conditions

All *Arabidopsis thaliana* plants used in this study were Columbia ecotype except where otherwise mentioned. Seeds of Arabidopsis were mutagenized by EMS and the M_2_ population was screened for the yellow leaf phenotype. Arabidopsis plants were grown on soil under controlled chamber conditions with a 16h light/8h dark photoperiod at 25 °C. Arabidopsis T-DNA homozygous lines of *Salk_095863* from the ABRC stock center were identified by PCR using gene-specific primer sets (see Supplementary Table S1 at *JXB* online).

For cold treatment, the surface-sterilized seeds were germinated and grown in Murashige and Skoog agar medium (4.3g l^−1^ Murashige and Skoog salts and 12g l^−1^ agar, pH 5.8) plates with 10g l^−1^ sucrose at 4 °C under light. After 6 weeks of growth, the cold-treated seedlings were photographed and analyzed.

### Pigment quantification and seedling weight measurement

Total chlorophyll from 4-week-old rosette leaves (25mg) was extracted with 80% acetone. The absorbance of the supernatants was measured at 645 and 663nm, and chlorophyll content was calculated by simultaneous equations as described by Inskeep and Bloom (1985). Carotenoids from leaf tissue of the same age (50mg) were extracted and analyzed as described previously (Li *et al.*, 2012). The samples were analyzed with three biological replicates.

For cold-treated seedling weight measurement, 15 seedlings from each line were combined and weighed. The analysis was performed with four biological replicates.

### Map-based cloning

An F_2_ mapping population was generated by crossing the *rps5* mutant in Columbia background with wild-type Arabidopsis ecotype Landsberg erecta. Genomic DNA from a total of 1258 homozygous *rps5* F_2_ plants was isolated and used for fine mapping. Linkage analysis was conducted using simple sequence length polymorphism markers based on the TAIR database (http://www.arabidopsis.org/) and Arabidopsis Mapping Platform (http://amp.genomics.org.cn/).

### Plasmid construction and plant transformation

For molecular complementation of the *rps5* mutant, a full-length *RPS5* (At2g33800) cDNA was amplified by PCR and cloned into pCAMBIA1300s (Zhou *et al.*, 2011) to generate the overexpression plasmid *35S:RPS5*. A native promoter complementation construct of *RPS5:RPS5* was generated, which carried 2.6kb genomic DNA including the native promoter, 5ʹ-untranslated region (UTR), coding region, 3ʹ-UTR, and 3ʹ flanking sequences of *RPS5*. The PCR amplification primer sets used are shown in Supplementary Table S1. All the constructs were confirmed by sequencing.

The plasmids were introduced into *Agrobacterium tumefaciens* strain GV3101 by electroporation and transformed into Arabidopsis plants by floral dip. Positive transgenic plants were screened on Murashige and Skoog plates containing 30mg l^−1^ carbenicillin and 50mg l^−1^ hygromycin (Yuan *et al.*, 2015).

### Subcellular localization

Full-length cDNAs of *RPS5* and *rps5* were cloned into the pGPTVII.GFP vector (Walter *et al.*, 2004) and electroporated into *A. tumefaciens* strain GV3101. *Agrobacterium* cells carrying the *35S:RPS5-GFP* or *35S:rps5-GFP* construct were infiltrated into 4-week-old tobacco leaves for transient expression. After infiltration, leaves of transgenic tobacco expressing RPS5-GFP and rps5-GFP fusion proteins were analyzed by a Leica TCS SP5 laser scanning confocal microscope as described by Zhou *et al.* (2015). The primers used for the plasmid construction are listed in Supplementary Table S1.

### RNA isolation and qRT-PCR

Total RNA from 4-week-old wild-type, *rps5*, and *Salk_095863* rosette leaves was extracted and used for cDNA synthesis with random hexamer primers as described by Zhang *et al.* (2015). Quantitative real-time RT-PCR (qRT-PCR) was performed using iTaqTM Universal SYBR Green Supermix (Bio-Rad) with gene-specific primer sets (Supplementary Table S1). Thermal cycling conditions consisted of a ﬁrst step of denaturation at 95 °C for 10min, followed by 40 cycles of denaturation for 15s at 95 °C and annealing/extension for 1min at 60 °C. Relative expression levels were calculated using the ^△△^Ct method (Lyi *et al.*, 2007) and normalized ﬁrst with a ubiquitin control and then with the expression of wild-type controls. Each sample was quantified in triplicate with three biological replicates.

### RNA gel blotting

Total RNA (3 μg) from 4-week-old leaves was separated on 1.5% denaturing agarose gels containing 2.2M formaldehyde, transferred to a positively charged nylon membrane (Hybond-N, Amersham), and fixed by UV crosslinking. 16S, 18S, 23S, 5S, and 4.5S rRNA probes were amplified by PCR using gene-speciﬁc primers as described by Yu *et al.* (2008). ^32^P-labeled cDNA probes were generated using a random primer DNA-labeling kit (Life Technologies) following the manufacturer’s protocol. Filters were pre-hybridized for 1h at 42 °C using NorthernMax prehybridization/hybridization buffer (Life Technologies), hybridized with ^32^P-labeled DNA probes overnight at 42 °C, and washed as described by Lyi *et al.* (2005). Storm 860 PhosphorImager (Molecular Dynamics) was used to detect the signals. The intensity of the image bands was quantified using ImageJ (http://www.di.uq.edu.au/sparqimagejblots).

### Proteomics analysis

Total proteins from 4-week-old rosette leaves of wild-type and *rps5* (Supplementary Figure S1) were extracted using the phenol extraction method (Yang *et al.*, 2007). Isobaric tags for relative and absolute quantiﬁcation (iTRAQ)-based proteomic analysis including protein purification, digestion, iTRAQ labeling, protein identification, and quantification was performed following methods detailed previously (Yang *et al.*, 2011). The iTRAQ-labeled samples were analyzed on Orbitrap Elite using a two-dimensional (2D) LC-MS/MS approach. In addition, a gel-based proteomic analysis including 2D gel electrophoresis, comparative 2D gel image analysis using SameSpots software (Nonlinear Dynamics), in-gel digestion, and 2DGeLC sample analysis on Synapt HDMS coupled with nan-Acquity was carried out as described (Wang *et al.*, 2013; Yang *et al.*, 2007, 2011). Proteins extracted from *RPS5*-overexpressing lines were analyzed on Orbitrap Elite using a label-free approach and the relative quantification of PRS5 was determined by extracted ion chromatograms as described by Vaidya *et al.* (2013). The peak areas of detected precursor ions at each specific *m/z* corresponding to the targeted peptides were obtained with mass tolerance at 5ppm using Xcalibur 2.2 software.

The functional classification of identified proteins was conducted based on the bincodes of MapMan (http://mapman.gabipd.org/web/guest/mapman). Protein subcellular localizations were predicted by TAIR10 (https://www.arabidopsis.org/) and the PPDB database (http://ppdb.tc.cornell.edu/).

## Results

### The *rps5* mutant shows pale yellow inner leaves and a developmental defect

**Fig. 1.**
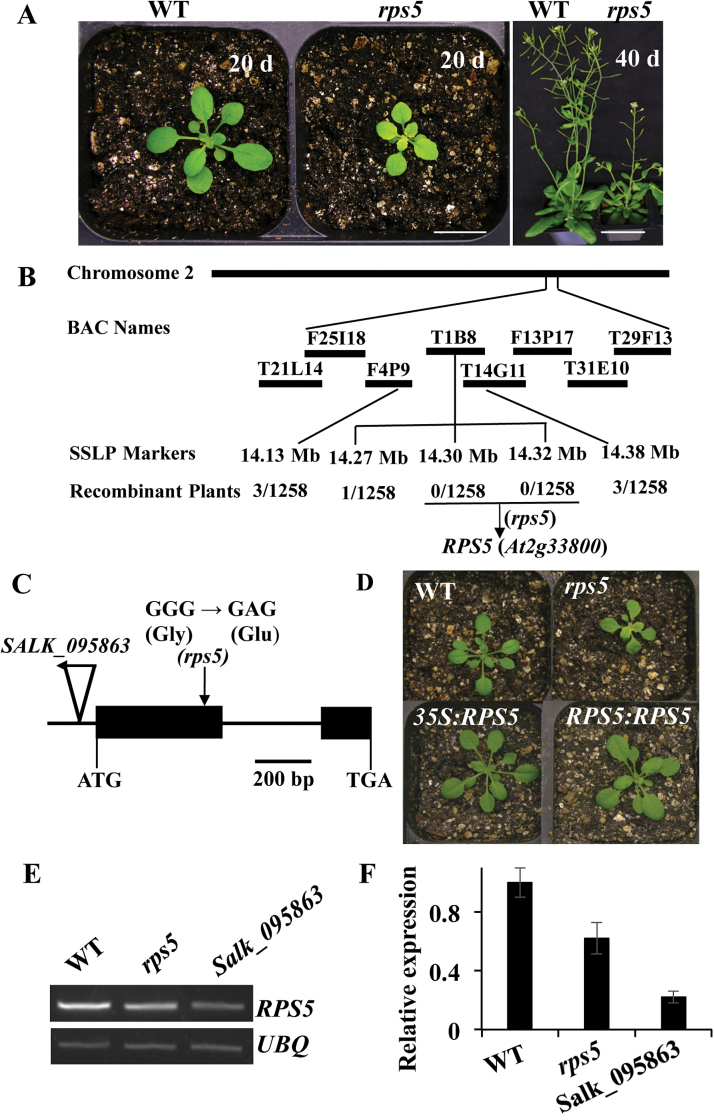
Molecular cloning of *rps5*. (A) Growth phenotype of wild-type and *rps5* mutant plants at 20 and 40 days old, respectively. Scale bars=1cm. (B) Positional cloning of *rps5*. Homozygous *rps5* F_2_ seedlings from a total of 1258 plants were used for fine mapping. (C) *RPS5* gene structure and the locations of the missense mutation for *rps5* and T-DNA insertion for *Salk_095863.* Exons and introns are shown as solid boxes and lines, respectively. The T-DNA insertion is indicated with a triangle and the single nucleotide change in *rps5* is indicated with a vertical arrow. (D) Complementation of the *rps5* mutant. The *rps5* phenotype was complemented with *RPS5* cDNA (*35S:RPS5*) or genomic DNA (*RPS5:RPS5*). Plants were 4 weeks old. (E, F) Expression of *RPS5* in wild-type and *rps5* plants detected by semi-quantitative RT-PCR and qRT-PCR, respectively. Data represent means±SD from three biological replicates with three technical repeats. (This figure is available in colour at *JXB* online.)

In an effort to identify genes involved in chloroplast development and pigment synthesis, we screened an EMS-mutagenized M_2_ population for mutants with abnormal leaf color. This screen uncovered a pale yellow inner leaf color mutant, *rps5,* whose phenotype was controlled by a single nuclear recessive gene. In comparison with wild-type plants, this mutant had pale yellow inner young leaves (Fig. 1A). The yellow color was distinct at the seedling stage and gradually became pale green during plant growth. In addition, a delay of 2 days in first true leaf emergence in the mutant plants was observed.

It is well established that pale green leaves are associated with impaired photosynthetic performance, which subsequently affects plant growth and development (Romani *et al.*, 2012; Tiller *et al.*, 2012). Consistent with this, the total chlorophyll and carotenoid levels were significantly lower in *rps5* than those in the wild-type plants (Supplementary Figure S2). The mature *rps5* mutant plants had a reduced growth rate and were much smaller than wild-type plants (Fig. 1A).

### The *rps5* mutant results from a missense mutation of ribosomal protein S5 (*RPS5*)

To isolate the gene responsible for *rps5*, a map-based cloning strategy was employed following the generation of an F_2_ mapping population by crossing *rps5* in Columbia ecotype with wild-type in Landsberg erecta background. A total of 1258 homozygous *rps5* mutant plants were selected and used for fine mapping. The *rps5* locus was mapped to the long arm of chromosome 2 between BACs T21L14 and T29F13 using simple sequence length polymorphism markers from the TAIR database and Arabidopsis Mapping Platform, and further narrowed to BAC clone T1B8 with two co-segregation markers (Fig. 1B). Thirteen candidate genes between the 14.30Mb and 14.32Mb regions on chromosome 2 from the *rps5* mutant were sequenced. A missense mutation was found in At2g33800 due to a G to A base substitution in the first exon in *rps5*, resulting in an amino acid Gly to Glu change (Fig. 1C). At2g33800 encodes a RP S5 family protein (RPS5). Thus, the mutant was named as *rps5.*

To determine whether this missense mutation in *RPS5* was responsible for the mutant phenotype of *rps5*, a complementation test was performed in *rps5* using the wild-type *RPS5* cDNA under control of the cauliﬂower mosaic virus 35S promoter. Over 20 positive transgenic lines overexpressing *RPS5* were obtained. *RPS5* restored the *rps5* mutant defects and the transgenic lines showed a completely wild-type phenotype (Fig. 1D). To further validate the cloned gene, we also performed a complementation test using the genomic DNA fragment containing the wild-type *RPS5* coding region and the associated flanking sequences. The genomic DNA construct was introduced into the *rps5* mutant. Again, the positive transgenic lines with endogenous promoter also displayed the wild-type phenotype (Fig. 1D). These results indicated that *RPS5* was responsible for the mutant phenotypes.

To determine whether the point mutation in *rps5* affected the transcript level of *RPS5*, its expression in 4-week-old plants was analyzed by semi-quantitative RT-PCR (Fig. 1E) and qRT-PCR (Fig. 1F). A *Salk_095863* line that carries a T-DNA insertion in the 5ʹ-UTR of *RPS5* (Fig. 1C) was also included. The Salk_095863 mutant showed growth and developmental defects, with smaller plants than *rps5* (Supplementary Figure S3), showing allele variation. Interestingly, the expression analyses revealed that the transcript level of *RPS5* in *rps5* was decreased in comparison with wild-type plants, although *rps5* was a missense mutation. Missense mutations have also been reported to affect gene expression in other studies (Takano *et al.*, 2010; Wang *et al.*, 2015). As expected, the *Salk_095863* line exhibited low transcript levels.

### RPS5 structure and subcellular localization

**Fig. 2.**
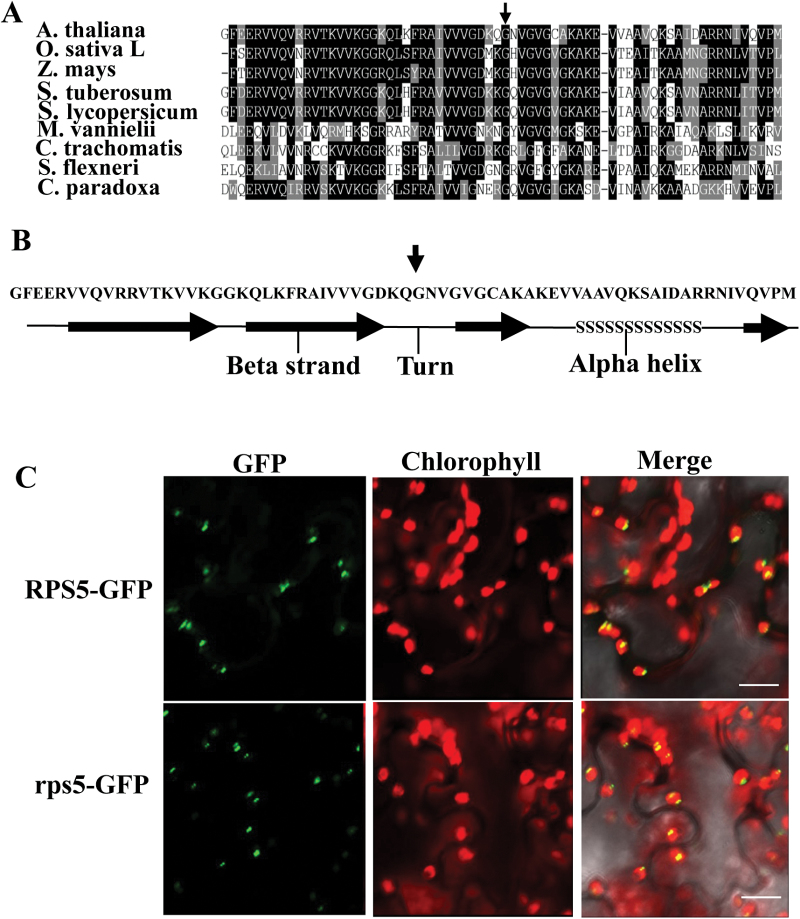
Partial sequence alignment, secondary structure prediction, and subcellular localization of RPS5. (A) N-terminal domain sequence alignment of RPS5 from Arabidopsis (*A. thaliana,* AEC08888), rice (*Oryza sativa*, NP_001050474), maize (*Zea mays,* NP_001150762), potato (*Solanum tuberosum,* XP_006347020), tomato (*Solanum lycopersicum,* XP_004232896*), Methanococcus vannielii* (P14036), *Chlamydia trachomatis* (P0A4C8), *Shigella flexneri* (P0A7W6), and *Cyanophora paradoxa* (P23402). The vertical arrow indicates the mutation site. (B) Predicted secondary structure of the RPS5 N-terminal domain. The vertical arrow indicates the Glu substitution site in the mutant protein. (C) Wild-type RPS5 (top) and mutant rps5 (bottom) are localized in chloroplasts of 4-week-old tobacco leaves transiently expressing GFP fusion proteins. Bars=25 µm. (This figure is available in colour at *JXB* online.)

RPS5 is a component of the chloroplast ribosome 30S small subunit (Yamaguchi *et al.*, 2000). The molecular mass of RPS5 mature protein was 32.6 kD. A sequence similarity search showed that only one *RPS5* gene orthologous to the *Escherichia coli* S5 protein gene existed in the Arabidopsis genome (Supplementary Table S2). Interestingly, the N-terminal region of RPS5 proteins was highly conserved among plants and bacterial species (Fig. 2A). The secondary structure for the N-terminal sequence and a three-dimensional 3D structure model for RPS5 protein were constructed using the Phyre2 server (http://www.sbg.bio.ic.ac.uk/phyre2/html/page.cgi?id=index; Kelley and Sternberg, 2009). The predicted secondary structure revealed that the amino acid substitution of RPS5 from *rps5* was located in the turn element between two beta strand sheets (Fig. 2B). We also predicted the 3D structures of RPS5 from wild-type and mutant plants using the Phyre2 server. The same 3D structures were observed (Supplementary Figure S4). One explanation for this result is that the software prediction was based on published data and the mutant site might not have been analyzed.

RPS5 protein was predicted to be localized in chloroplasts (http://www.cbs.dtu.dk/services/TargetP/; Emanuelsson *et al.*, 2007). To validate this, we investigated the subcellular location of RPS5 by transiently expressing the *RPS5-GFP* construct in tobacco leaves. The fusion protein signal was monitored by confocal microscopy. Merging the chlorophyll autoﬂuorescence and green ﬂuorescence showed that the signals were clearly observed in chloroplasts (Fig. 2C), consistent with the previous indication of a chloroplast thylakoid membrane location in a chloroplast subproteome analysis (Friso *et al.*, 2004).

Plastid localization can be altered by single amino acid changes (Shumskaya *et al.*, 2012). We investigated whether the single amino acid substitution of RPS5 could affect the localization of RPS5. The *rps5-GFP* construct was infiltrated into tobacco leaves and expression of the fusion protein was examined. The signal pattern of rps5-GFP fusion protein was identical to that of RPS5-GFP (Fig. 2C). These results showed that the *RPS5* mutation did not alter the subcellular location of the protein.

### *RPS5* mutation significantly reduces the efficiency of 16S rRNA processing

**Fig. 3.**
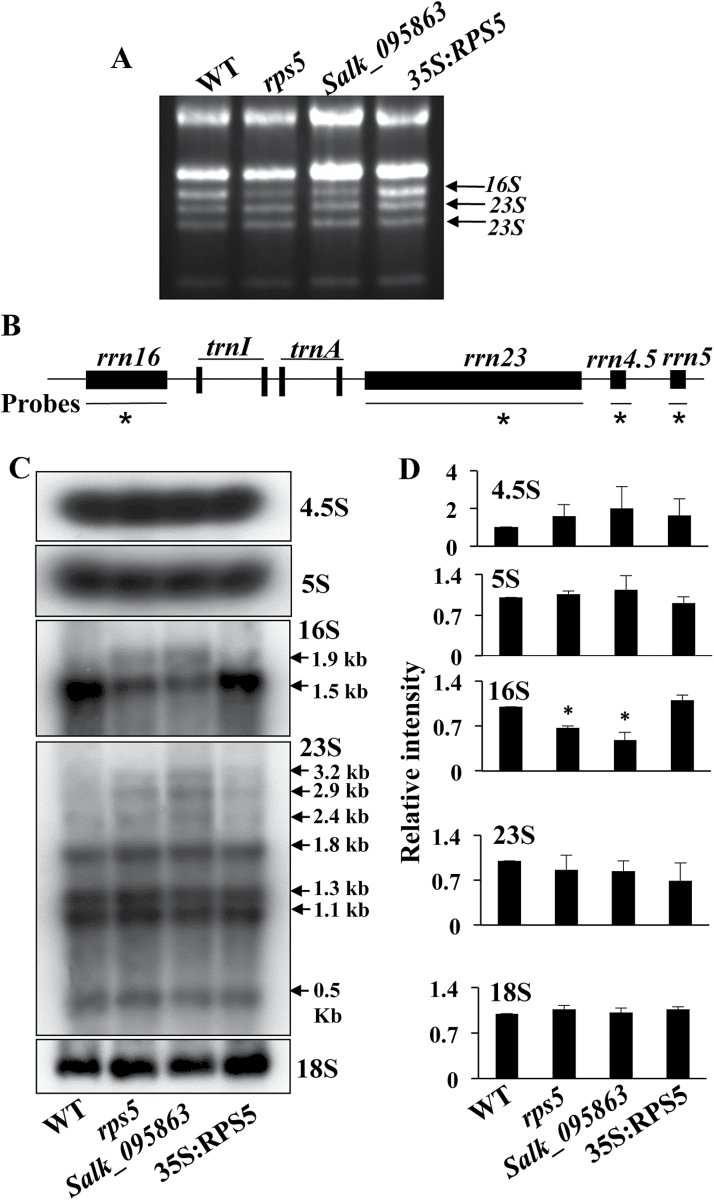
Chloroplast rRNA accumulation and processing in wild-type (WT), mutant lines (*rps5* and *Salk_095863*), and a complementation *RPS5*-overexpressing line (*35S:RPS5*). (A) rRNA accumulation pattern in an ethidium bromide-stained gel. The mutant lines had reduced levels of 16S rRNA, identified based on Kleinknecht *et al.* (2014) and Yu *et al.* (2008). (B) Schematic diagram of the chloroplast *rrn* operon in Arabidopsis. The probes used for northern blot analysis are marked by black lines under the operon with asterisks. (C) Northern blot analysis of chloroplast 4.5S, 5S, 16S, 23S, and cytoplasmic18S rRNAs. Total RNA (3 µg) from 4-week-old leaves was used for northern blot experiments. Equal loading controls for northern blot analysis are shown in Supplementary Figure S5. (D) Quantification of the mature bands in C using ImageJ (http://www.di.uq.edu.au/sparqimagejblots). Asterisks in 16S indicate significant differences (*p*<0.05).

To investigate whether mutation in the plastid RPS5 would impair chloroplast 16S rRNA processing, we initially examined the pattern of total RNA by running denaturing agarose gels and staining them with ethidium bromide. As shown in Fig. 3A, chloroplast 16S rRNA was decreased in the *rps5* and *Salk_095863* mutants relative to wild-type plants. The reduction could be rescued by overexpressing *RPS5* in the *rps5* mutant plants. These results indicated that RPS5 had an effect on 16S rRNA processing.

Plastid rRNAs include 23S, 16S, 5S, and 4.5S, which form part of the chloroplast *rrn* operon (Fig. 3B) (Kleinknecht *et al.*, 2014; Yu *et al.*, 2008). These rRNAs are transcribed to yield large RNA precursors that are finally processed to mature rRNAs by a series of processing steps (Harris *et al.*, 1994). To see whether RPS5 mutation affected chloroplast rRNA processing, the plastid rRNA accumulation patterns were analyzed in detail by RNA gel blots using gene-speciﬁc probes (Fig. 3B). No signiﬁcant differences in the abundance of 4.5S and 5S sRNA were observed between *rps5* or *Salk_095863* and wild-type plants (Fig. 3C, D). In agreement with the results from the ethidium bromide-stained denaturing agarose gels (Fig. 3A), the quantity of mature 16S rRNA in the *rps5* and *Salk_095863* mutants was dramatically reduced to 65% and 50%, respectively, in comparison with the wild-type control (Fig. 3D), whereas the 16S rRNA 1.9kb precursors were increased in the mutant plants (Fig. 3C). The 16S rRNA processing deficiency in *rps5* was rescued by overexpression of *RPS5* (Fig. 3C, D). In addition, a slight increase of the 3.2, 2.9, and 2.4kb precursor rRNAs was observed in the *rps5* mutants relative to wild-type plants using a 23S rRNA gene-specific probe (Fig. 3C). Cytosolic 18S rRNA was also examined. As expected, similar 18S rRNA levels were observed in these mutant and wild-type plants (Fig. 3C, D), indicating that the *RPS5* mutations did not affect cytosolic rRNA accumulation. The loading controls of RNA gel blots are shown in Supplementary Figure S5. Taken together, these data revealed that *RPS5* mutations mainly caused the impairment of chloroplast 16S rRNA processing.

### Comparative proteomics analysis reveals the role of RPS5 in affecting photosystem I and II core components and plastid RPs

**Fig. 4.**
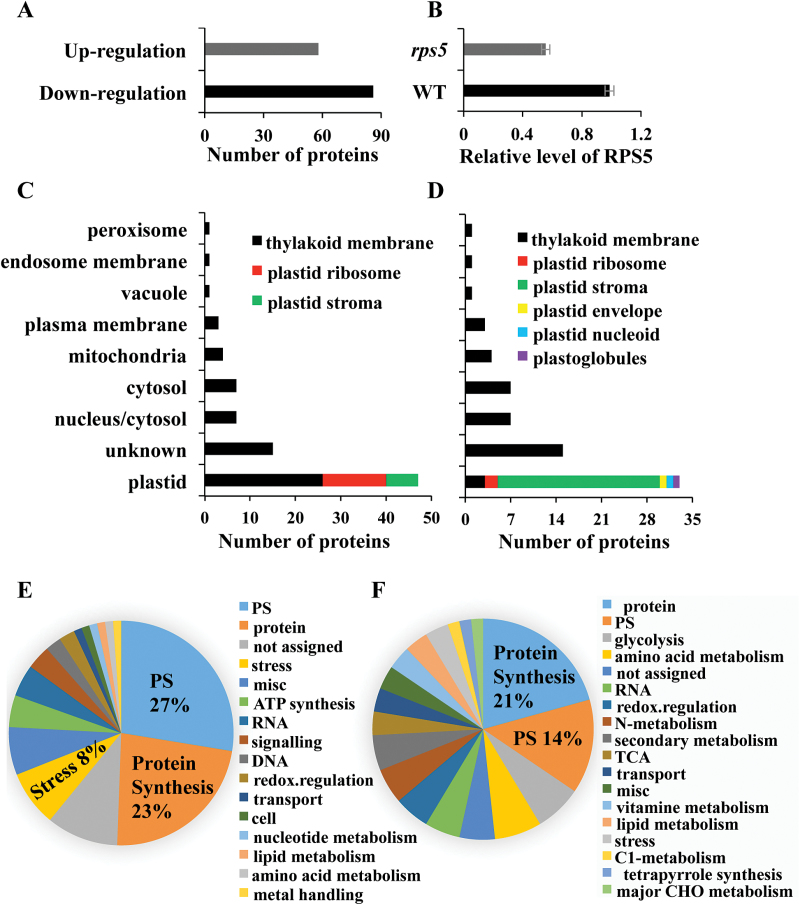
Comparative proteomic analysis of the differentially expressed proteins in the *rps5* proteome. (A) Numbers of up- and down-regulated proteins showing significant differences between the wild-type and *rps5* proteome. (B) Relative level of RPS5 protein in wild-type and *rps5* plants. Values are means±SD from three biological replicates. (C, D) Subcellular localization of (C) down-regulated and (D) up-regulated proteins in the *rps5* proteome. (E, F) Functional classification of (E) down-regulated and (F) up-regulated proteins in the *rps5* proteome. (This figure is available in colour at *JXB* online.)

While the effects of RP defects on a number of plastid and nuclear proteins have been examined previously via immunological analysis (Romani *et al.*, 2012; Tiller *et al.*, 2012), there is limited information for their global effects on protein synthesis. Impaired chloroplast 16S rRNA processing can affect chloroplast translation (Kleinknecht *et al.*, 2014). To obtain a global and quantitative view of proteins altered in *rps5*, a comparative proteomic analysis of total proteins from 4-week-old wild-type and *rps5* leaves was carried out using an iTRAQ-based method. A total of 1007 proteins were identiﬁed, and 294 proteins were differentially expressed (*P*<0.05) (Supplementary Table S3). Among the differentially expressed proteins, 58 proteins were up-regulated with at least 1.33-fold change and 86 proteins were down-regulated with 0.75- or less fold change in all three biological replicates of *rps5* (Fig. 4A; Supplementary Table S4). Consistent with the reduced *RPS5* expression (Fig. 1E, F), the RPS5 protein level was also detected to have ~43% reduction in *rps5* compared with wild-type plants (Fig. 4B).

The majority of the differentially regulated proteins, in either the down-regulated (Fig. 4C) or up-regulated (Fig. 4D) groups, were localized to plastid. Notably, among the down-regulated plastid proteins, over half (26 of 47) were thylakoid membrane proteins (Fig. 4C). In contrast, a majority (25 of 33) of up-regulated plastid proteins were stroma proteins (Fig. 4D). These differentially expressed proteins were categorized into functional groups using MapMan (http://mapman.gabipd.org/web/guest/mapman). Proteins associated with photosynthesis and protein synthesis represented the most abundant groups (Fig. 4E, F; Supplementary Tables S5 and S6), revealing that the defect in RPS5 exerted profound effects on chloroplast-localized proteins involved in photosynthesis and protein synthesis.

**Fig. 5.**
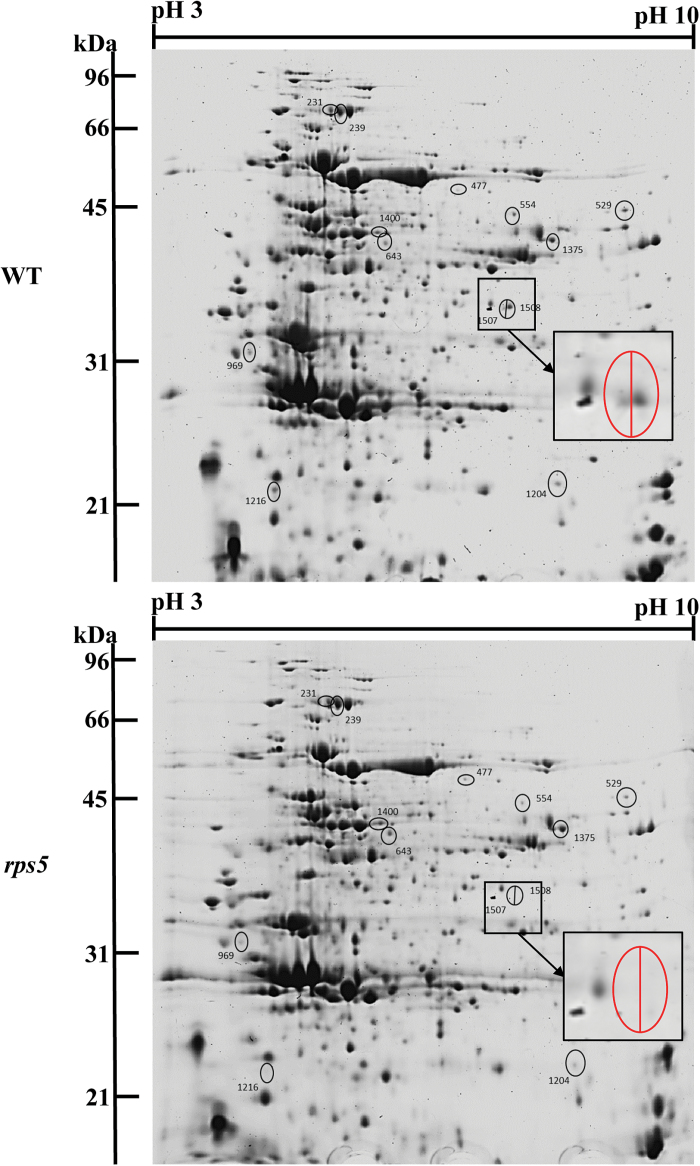
2D gel-based proteomic analysis of wild-type (top) and *rps5* (bottom) plants. The differentially expressed proteins in wild-type and mutant plants are labelled by black circles and the enlarged 1507/1508 spots are indicated in red circles. Spots 1507/1508 indicate RPS5 protein. (This figure is available in colour at *JXB* online.)

In addition, to provide another approach examining the differentially expressed dominant proteins and validating iTRAQ data, a 2D gel-based quantitative proteomic analysis was conducted using SameSpots image analysis software. Thirteen spots with marked differences between wild-type and *rps5* were selected to identify the differentially expressed proteins (Fig. 5). Consistent with the iTRAQ results, seven proteins, including RPS5, photosystem II subunit R (PsbR), ATPase subunit b (PDE334), and Rubisco small chain 1A (RBCS1A), were decreased in *rps5*, whereas the other seven proteins, including fructose-bisphosphate aldolase 5 (FBA5) and transketolase (ATTKL1), were increased in the mutant plants compared with wild-type plants (Table 1). The cross-validation of these differentially expressed proteins by 2D gel-based proteomic analysis corroborated the iTRAQ results and further confirmed that the amount of RPS5 protein was greatly reduced in *rps5* plants relative to wild-type plants (spot 1507/1508 in Fig. 5; Table 1).

**Table 1.** Cross-validation of proteins identified by iTRAQ- and 2D gel-based proteomic analyses

Gene locus	Protein description	emPAI ratio*	SC_unNor ratio*	Spots	iTRAQ ratio*
AT1G79040.1	Photosystem II subunit R	0.23	0.5	529	0.55
AT2G33800.1	Ribosomal protein S5	0.05	0.03	1508/1507	0.56
AT1G64510.1	Ribosomal protein S6	0.57	0.8	1216	0.6
AT4G32260.1	ATPase subunit b	0.55	0.7	1216	0.61
AT3G52150.1	Plastid-specific ribosomal protein 2	0.63	0.7	1204	0.66
AT1G67090.1	Ribulose bisphosphate carboxylase small chain 1A	0.41	0.7	969	0.68
AT5G07020.1	Proline-rich protein family	0.14	0.3	969	0.73
AT1G11860.1	Glycine cleavage T-protein	2.5	1.6	1375	1.33
AT5G26000.1	Myrosinase 1	4.2	4	239/231	1.38
AT2G39730.1	Rubisco activase	2.2	1.4	1400	1.48
AT1G23310.1	Glutamate:glyoxylate aminotransferase	2.2	1.8	477	1.54
AT5G43940.1	Alcohol dehydrogenase class-3	1.34	2.5	554	1.4
AT4G26530.1	Fructose-bisphosphate aldolase 5	2.6	1.9	231	1.66
AT3G60750.1	Transketolase	2.7	1.6	231	1.82

emPAI ratio, protein ratio calculated by Exponentially Modified Protein Abundance Index; SC_unNor ratio, protein ratio calculated by Spectrum Counting_unNormalization. * The protein ratio is *rps5*:wild-type.

#### Down-regulated proteins in rps5

Among the 86 down-regulated proteins (Supplementary Table S4), numerous proteins (28%) were involved in photosynthesis (Fig. 4E; Supplementary Table S5). Many of these photosynthetic proteins were localized in thylakoid membrane, the site of the light-dependent reactions of photosynthesis. They included six photosystem I polypeptide subunits (PSAD-1, PSAD-2, PSAE-1, PSAN, PSAL, and PSAF), 11 photosystem II polypeptide subunits (PSBTn-1, PSBTn-2, PSBR, psbE, psbH, PSBP-1, PSBP-2, PSBO-1, PSBO-2, PSBQ-1, and PSBQ-2), and three ATP synthases (ATPG, atpA, and atpB) (Table 2). Four (psbE, psbH, atpA, and atpB) were chloroplast-encoded proteins. Proteins that function in the Calvin cycle, such as CP12 domain-containing protein and Rubisco small subunit-4 (RBCS-4), were also dramatically reduced in the *rps5* mutant. These results indicate that RPS5 exerts a strong effect on the photosynthetic proteins.

**Table 2.** Down-regulated proteins involved in photosynthesis and the chloroplast 30S subunit

Accession	Protein description	Ratio*	Accession	Protein description	Ratio*
*Proteins involved in photosynthesis*			*Ribosomal proteins in chloroplast 30S subunit*		
ATCG00710.1	Photosystem II reaction center protein H	0.36	AT4G34620.1	Ribosomal protein S16B	0.53
AT2G30790.1	Photosystem II subunit P-2	0.47	AT2G33800.1	Ribosomal protein S5	0.56
AT3G21055.1	Photosystem II subunit T	0.51	ATCG00900.1	Ribosomal protein S7B	0.57
AT1G06680.1	Photosystem II subunit P-1	0.55	ATCG01120.1	Ribosomal protein S15	0.58
AT1G79040.1	Photosystem II subunit R	0.55	AT1G79850.1	Ribosomal protein S17	0.58
AT1G51400.1	Photosystem II 5 kD protein	0.59	AT1G64510.1	Ribosomal protein S6	0.6
AT3G50820.1	Photosystem II subunit O-2	0.6	ATCG00770.1	Ribosomal protein S8	0.64
ATCG00580.1	Photosystem II reaction center protein E	0.6	ATCG00650.1	Ribosomal protein S18	0.65
AT1G76450.1	Photosystem II reaction center PsbP family protein	0.61	AT3G15190.1	Ribosomal protein S20	0.66
AT5G66570.1	PS II oxygen-evolving complex 1	0.61	AT1G74970.1	Ribosomal protein S9	0.67
AT4G05180.1	Photosystem II subunit Q-2	0.64	AT5G30510.1	Ribosomal protein S1	0.69
AT4G21280.1	Photosystem II subunit QA	0.65	ATCG00380.1	Ribosomal protein S4	0.71
AT1G03130.1	Photosystem I subunit D-2	0.47	AT5G14320.1	Ribosomal protein S13	0.71
AT4G28750.1	Photosystem I reaction centre subunit IV	0.48			
AT4G02770.1	Photosystem I subunit D-1	0.55			
AT5G64040.1	Photosystem I reaction center subunit N	0.61			
AT4G12800.1	Photosystem I subunit l	0.64			
AT1G31330.1	Photosystem I subunit F	0.74			
AT1G03130.1	Photosystem I subunit D-2	0.47			
AT4G28750.1	Photosystem I reaction centre subunit IV	0.48			
AT4G02770.1	Photosystem I subunit D-1	0.55			
AT5G64040.1	Photosystem I reaction center subunit N	0.61			
AT4G32260.1	Pigment defective 334	0.61			
ATCG00120.1	ATP synthase subunit alpha	0.68			
ATCG00480.1	ATP synthase subunit beta	0.73			
AT2G47400.1	CP12	0.66			
AT1G67090.1	Rubisco small subunit-4	0.68			

* The protein ratio is *rps5*:wild-type from iTRAQ analysis.

In addition, a high proportion (23%) of the down-regulated proteins were those involved in protein synthesis (Fig. 4E, Supplementary Table S5). Eighteen RPs were down-regulated in the *rps5* mutant in comparison to wild-type plants. Most of these (13 of 18) were involved in the chloroplast 30S subunit (Table 2), which might result from RPS5 being a component of the chloroplast 30S subunit. Low plastid RP levels could affect ribosome function and plastid translation, which are essential for plant development and photosynthesis (Horiguchi *et al.*, 2012; Romani *et al.*, 2012; Tiller and Bock, 2014).

#### Up-regulated proteins in rps5

A total of 58 proteins were up-regulated in *rps5*, 33 of which were localized to chloroplast (Supplementary Table S4; Fig. 4D). Among these plastid proteins, 25 were in the plastid stroma and mainly involved in protein synthesis, photosynthesis, and nitrogen metabolism (Fig. 4F; Supplementary Table S6). In addition, proteins involved in nitrogen metabolism, secondary metabolism, one-carbon metabolism, vitamin metabolism, tricarboxylic acid cycle, and glycolysis were found only among the up-regulated proteins (Supplementary Table S6).

### Transcript levels of photosynthesis-related genes are not affected in *rps5*

Comparative proteomic analysis indicated that many proteins involved in photosynthesis were altered in *rps5*. To assess whether the expression of the photosynthesis-related genes was affected in the *rps5* plants, the transcript levels of some nuclear- and plastid-encoded genes involved in photosynthesis were tested by qRT-PCR. The genes examined included those involved in photosystem I (*psaA*, *psaB*, *psaC*, *PSAD*, *PSAE*, and *PSAF*) and photosystem II (*psbA*, *psbB*, *psbC*, *psbD*, and *psbE*), as well as Rubisco large and small subunits (*RbcL* and *RBCS*). As shown in Supplementary Figure S6, no dramatic difference in expression of all the tested genes was observed in *rps5* or *Salk_095863* compared with wild-type plants. These data, along with the proteomics studies, indicate that the transcript profile of photosynthesis-related genes was not disturbed in the mutant plants and that RPS5 affected photosynthetic proteins at the translational level.

### Overexpression of RPS5 enhances cold tolerance

**Fig. 6.**
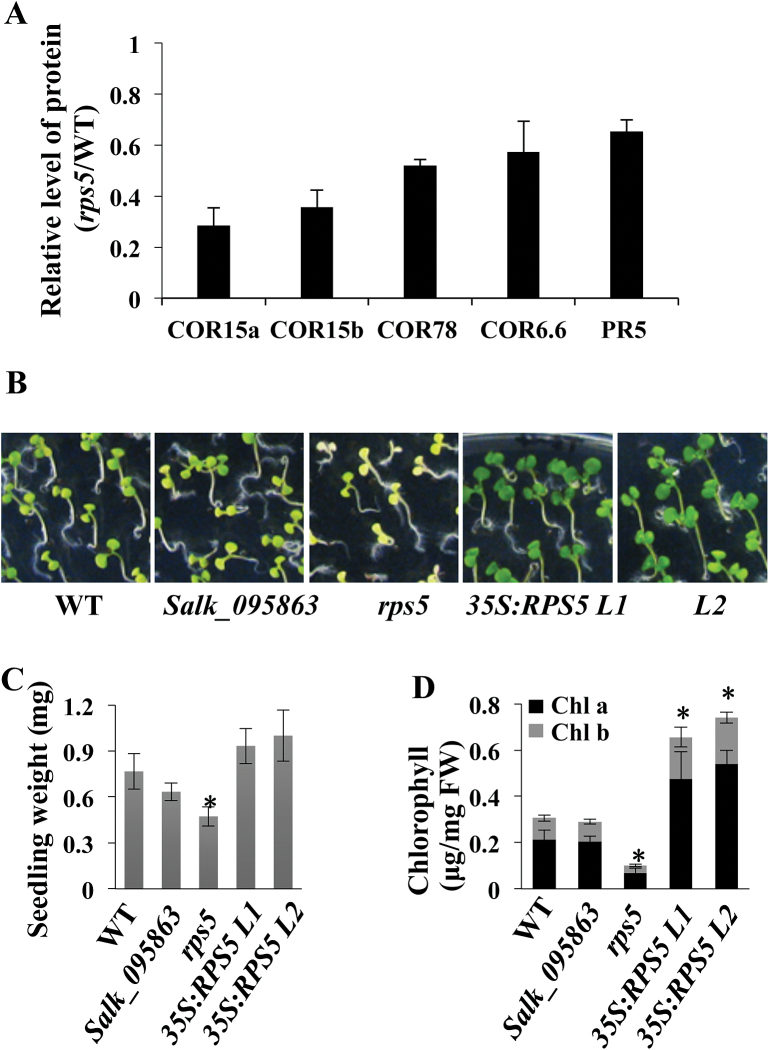
Role of RPS5 in cold stress tolerance. (A) Relative levels of proteins associated with the cold stress response in *rps5* plants. Values represent means±SD from three biological replicates. (B) Phenotypes of wild-type (WT), *Salk_095863, rps5*, and two *RPS5* overexpression lines grown on Murashige and Skoog medium at 4 °C for 6 weeks. (C) Average weight per seedling grown at 4 °C for 6 weeks. (D) Chlorophyll content of seedlings grown at 4 °C for 6 weeks. Values are means±SD from four biological replicates. (This figure is available in colour at *JXB* online.)

Apart from those proteins associated with photosynthesis and protein synthesis, interestingly, a number of proteins involved in stress responses were found in the comparative proteomic analysis to be reduced in the *rps5* mutant (Supplementary Table S4). These included four cold-regulated proteins (COR15a, COR15b, COR78, and COR6.6) and a pathogenesis-related protein (PR5) (Fig. 6A). In addition, two dehydrin family proteins (ERD10 and ERD14) and five glutathione transferases (GST9, GSTF2, GSTF10, GSTU19, and GSTU20) were reduced in the *rps5* mutant (Supplementary Table S4). The down-regulation of these stress-related proteins in *rps5* suggested a potential role of *RPS5* in stress responses.

To provide evidence in support of this, we compared plant growth of wild-type, *Salk_095863*, *rps5*, and two *RPS5*-overexpressing lines under cold treatment at 4 °C. The two *RPS5*-overexpressing lines contained 1.53- and 1.78-fold increases in the RPS5 protein levels, respectively, as measured via relative protein quantification by calculating the identified peptide peak areas of RPS5. When these plants were germinated and grown at 4 °C for 6 weeks, the seedlings of the two *RPS5*-overexpressing lines grew much better and had a dark green color in comparison with those of wild-type plants (Fig. 6B). In contrast, the seedlings of *rps5* showed a more yellow and less green color than the seedlings of wild-type control plants (Fig. 6B). The *Salk_095863* line had a weak phenotype in comparison with the *rps5* mutant (Fig. 6B). The difference in cold tolerance phenotypes between *Salk_095863* and *rps5* might be due to the different accumulation of functional RPS5 protein in *Salk_095863* and the formation of malfunctioning RPS5 protein in *rps5* due to the missense mutation. Noticeably, the *rps5* mutant appeared to be more severely affected in the cold but less affected under normal growth conditions than the *Salk_095863* line, suggesting that the malfunctioning RPS5 protein could function partially under normal growth conditions but was non-functional in cold conditions.

The seedling weight after 6 weeks of growth at 4 °C was measured. The *rps5* mutant exhibited significantly lower seedling weight than the wild-type control; in contrast, the two overexpressing lines showed enhanced seedling weight (Fig. 6C). Moreover, the chlorophyll content was also significantly decreased in the *rps5* mutant and increased in the two overexpressing lines (Fig. 6D). These data support the potential role of *RPS5* in cold stress tolerance.

## Discussion

Although a growing number of recent studies support the critical roles of RPs in plant growth and development (Byrne, 2009; Horiguchi *et al.*, 2012; Schippers and Mueller-Roeber, 2010; Tiller and Bock, 2014), it is not fully understood how they affect these processes. In addition, the functions of some individual RPs remain largely unknown. In this study we identified a mutant *rps5* with a missense mutation, which resulted in a Gly to Glu substitution in the RPS5 protein and led to a reduced level of RPS5 expression and possible malfunction of the RPS5 protein. The defect in RPS5 enabled us to analyze the roles of this protein. RPS5 is a component of the 30S subunit in chloroplasts and is highly conserved in all species (Ramakrishnan and White, 1992). RPS5 is suggested to be required for translation (Ramakrishnan and White, 1992). In plants, a very preliminary analysis of the *rps5* Salk line was briefly described in a large-scale reverse genetic screen (Bryant *et al.*, 2011). In this study, we show that RPS5 is important for plastid ribosome function and is involved in photosynthesis, plant development, and cold stress resistance in Arabidopsis.

*RPS5* is expressed at all stages of plant growth and especially highly in the rosette leaves (Supplementary Figure S7). Our data revealed that the *rps5* mutation reduced the accumulation of chloroplast 16S rRNA and impaired the processing of 16S rRNA. Our quantitative proteomic analysis showed that the mutation exerted a strong effect on the levels of many proteins involved in photosynthesis and protein synthesis. Such alterations and effects likely contributed to the mutant phenotypes of yellow leaves and overall reduced growth in *rps5* plants. The proteomic analysis also suggested that *RPS5* likely contributed to abiotic stress resistance, which was supported by the cold tolerance test.

### RPS5 is required for chloroplast 16S rRNA processing

Ribosome assembly affects plastid translation, which is essential for plant development and photosynthesis (Schippers and Mueller-Roeber, 2010; Tiller and Bock, 2014). The ribosome contains rRNAs, RPs, and accessory factors, and all ribosomal functions rely on rRNAs. Crystal structure analysis of the ribosome indicates the existence of complicated interactions among the ribosomal components (Yusupov *et al.*, 2001). rRNAs are highly abundant in cells and are easily observed on denaturing agarose gels. Changes in their abundance are often used as an indicator for factors affecting ribosomes (Tiller *et al.*, 2012; Walter *et al.*, 2010). Two plastid RPs (RPS17 and PSRP4) are suggested to be essential for the stability of the 30S ribosomal subunit, as the ratio of 16S rRNA:18S rRNA is greatly decreased and the ratio of 23S rRNA:18S rRNA remains unchanged in the *rps17* and *psrp4* mutants (Tiller *et al.*, 2012). In addition, a recent study shows that mutation in *RBF1*, a ribosome-binding factor, leads to defects in chloroplast 16S rRNA processing and reduced accumulation of the 30S ribosomal subunit in Arabidopsis (Fristedt *et al.*, 2014). We found an up to 50% reduction in 16S rRNA in *rps5* mutants (Fig. 3D), implying that assembly of the chloroplast 30S subunit was affected by RPS5 (Tiller and Bock, 2014).

Although bacterial RPS5 belongs to the tertiary binding protein in the 30S ribosome assembly map (Shajani *et al.*, 2011), the crystal structure of bacterial RPS5 reveals that its many sites interact with 16S rRNA (Ramakrishnan and White, 1992; Wimberly *et al.*, 2000). This observation provides a basis for the assumption that mutation in RPS5 could cause a defect in the 16S rRNA processing. Indeed, our RNA gel blotting data demonstrated the accumulation of 16S precursor and a much smaller quantity of the mature chloroplast 16S rRNA in *rps5* and *Salk_095863* mutants (Fig. 3C, D). The reduced processing of 16S rRNA in these mutants could result from the reduced level of *RPS5* mRNA, like the Salk T-DNA line. It could be also due to the Gly to Glu mutation in the turn element between two beta strand sheets of RPS5 protein, which might also affect the interaction between RPS5 and 16S rRNA and the normal function of RPS5.

RPs have been shown to exert distinctive effects on rRNA processing in chloroplasts, and not all plastid RPs alter rRNA processing. The abundance of 23S and 16S rRNA precursors is greatly increased in the *rps17* and *psrp4* mutants, and the accumulation of 4.5S and 5S rRNAs is greatly reduced in *psrp3* (Tiller *et al.*, 2012). However, the abundance of chloroplast rRNA and the amount of rRNA processing are not changed in the *psrp2* and *psrp6* mutants (Tiller *et al.*, 2012). RPs may have different active sites with which to interact with different rRNAs at specific loci, resulting in plastid RPs having different effects on rRNA processing and abundance (Ramakrishnan, 2002).

### Global suppression of specific groups of protein synthesis is responsible for the *rps5* growth phenotypes

Comparative quantitative proteomics is a powerful tool to discover the proteins and pathways that are associated with or affected by specific factors and processes. As the effects of RPs on protein synthesis at global level have been subjected to limited studies, we performed comparative proteomic analysis and identified the differentially expressed proteins between wild-type and *rps5* plants. Proteomic data by both iTRAQ and 2D gel-based analyses revealed that RPS5 was decreased by approximately 50% in *rps5* (Fig. 4B; Supplementary Table S4). RPS5 was found to exert a profound effect on a group of both plastid genome-encoded and also nucleic genome-encoded plastid proteins. Since *rps5* causes a defect in chloroplast development, this defect might in turn affect distinctive sets of target nuclear gene expression and then protein levels via retrograde plastid-to-nucleus signaling (Chi *et al.*, 2013) to affect the expression of nucleic genome-encoded proteins.

A majority of the differentially regulated proteins were located in plastids. One large group of these down-regulated plastid proteins was associated with photosynthesis (Fig. 4E; Supplementary Table S5). Many proteins involved in photosystems I and II polypeptide subunits were reduced in *rps5* (Table 2). The reduction of these core components of photosynthetic proteins explained the yellow leaf and growth defect phenotypes observed in *rps5*. Indeed, a decrease in the accumulation of several photosynthetic proteins (i.e. psaA, PSAD, PSAF, psbB, Lhca2, and/or rbcL) has been reported to be the cause of defects in photosynthetic performance due to reduced translational capacity (Romani *et al.*, 2012; Tiller *et al.*, 2012).

Another large group of down-regulated plastid proteins in *rps5* was plastid RPs, including plastid RP S13, S17, and S20 (Table 2), some of which are known to be associated with growth and developmental defects. PRPS13 and PRPS20 are required for embryo development in Arabidopsis (Bryant *et al.*, 2011; Romani *et al.*, 2012). Similarly, a defect in rice PRPS20 results in albino and seedling-lethal phenotypes (Gong *et al.*, 2013). Mutation in PRPS17 leads to extended leaf longevity and reduced leaf growth rate (Woo *et al.*, 2002). The combination effect of decreased levels of multiple plastid RPs likely caused the phenotypic behavior of *rps5.*

In addition, most (13 of 18) of those down-regulated RPs were components of the chloroplast 30S subunit. These proteins are known to interact with 16S rRNA (Stern *et al.*, 1989; Wimberly *et al.*, 2000). Down-regulation of a large number of plastid RPs most likely seriously impaired the assembly of the chloroplast ribosome, subsequently reducing plastid translation and affecting overall plant development.

### RPS5 expression affects cold stress tolerance

Interestingly, comparative proteomics also identified that some proteins that participate in abiotic stress responses were dramatically decreased in *rps5* (Fig. 6A; Supplementary Table S4). These proteins are encoded by nuclear genes and their expression was down-regulated in *rps5,* which likely resulted from retrograde plastid-to-nucleus signaling due to the defect in chloroplasts in *rps5* (Chi *et al.*, 2013). Four cold-regulated proteins (COR6.6, COR15A, COR15B, and COR78) (Thomashow, 1998) were included among the down-regulated proteins; the suppression of their expression in *rps5* suggested that RPS5 might play a role in cold stress responses.

Most of the RPs are associated with development, and only a few studies have reported the association of RPs with stress responses. Chloroplast-localized RPS1 protein has been found to be associated with the heat stress response, and knock-down of *RPS1* inhibits the expression of HsfA2-dependent heat-stress responses in Arabidopsis (Yu *et al.*, 2012). Tobacco RPL33, a non-essential plastid-encoded RP, has been shown to play a critical role in plant recovery from chilling stress (Rogalski *et al.*, 2008). Here, we found that *rps5* showed reduced plant cold tolerance, with significantly decreased seedling weight and chlorophyll content, whereas *RPS5*-overexpressing plants exhibited enhanced plant cold tolerance, with increased seedling weight and chlorophyll content, suggesting a potential role of RPS5 in stress tolerance. Noticeably, although *rps5* with the missense mutation exhibited higher RPS5 expression than *Salk_095863*, *rps5* exhibited significantly less cold tolerance than *Salk_095863*. This result indicates that the Gly to Glu substitution of RPS5 protein in *rps5* produced a malfunctioning protein, contributing to the cold sensitivity phenotype, whereas the low accumulation of functional RPS5 protein in *Salk_095863* enabled the plants to better adapt to cold than *rps5* plants.

Together, our results show that mutation in RPS5 causes impaired processing of 16S rRNA, the key component of the 30S ribosomal subunit. Mutation of RPS5 greatly affected the expression of core components of photosystem I and photosystem II, and also greatly influenced the level of plastid RPs in the 30S ribosomal subunit. Because both plastid rRNAs and RPs are important components of the chloroplast ribosome, chloroplast 30S subunit assembly was likely affected, resulting in the mutant phenotypes of pigment deficiency and development defect. In addition, our results suggested a role of RPS5 in cold stress tolerance, possibly via a reduced plastid translational capacity.

## Supplementary data

Figure S1. The phenotype of 4-week-old wild-type and *rps5* mutant plants, and gel evaluation of proteins used for proteomic analysis.

Figure S2. Pigment content of wild-type and the *rps5* mutant.

Figure S3. The growth phenotype of wild-type, *rps5*, and *Salk_095863* mutants.

Figure S4. RPS5 3D structure model.

Figure S5. RNA gel blotting loading controls.

Figure S6. Expression of photosynthesis-related genes in wild-type, *rps5*, and *Salk_095863*.

Figure S7. Gene expression pattern of RPS5 based on Arabidopsis e-FP Browser.

Table S1. Primers used in this study.

Table S2. Blast analysis of *RPS5* in the Arabidopsis genome.

Table S3. List of 294 proteins showing significant difference (*p*<0.05) detected by iTRAQ proteomic analysis.

Table S4. Proteins significantly down- and up-regulated in *rps5* compared with wild-type by iTRAQ proteomic analysis.

Table S5. Detailed information of functional classification of the down-regulated proteins in *rps5* proteome.

Table S6. Detailed information of functional classification of up-regulated proteins in *rps5* proteome.

Supplementary Data

## List of abbreviations:

2D: two-dimensional
3D: three-dimensional
EMS: ethyl methanesulfonate;
iTRAQ: isobaric tags for relative and absolute quantiﬁcation
qRT-PCR: quantitative real-time RT-PCR
RP: ribosomal protein;
UTR: untranslated region.
