# Supplementary material for: Plastid ribosomal protein S5 is involved in photosynthesis, plant development, and cold stress tolerance in Arabidopsis
Source: J Exp Bot. 2016 Mar 22;67(9):2731–44. doi: 10.1093/jxb/erw106 (PMC4861020; doi:10.1093/jxb/erw106)
Supplement: Supplementary Data [file supp_67_9_2731__index.html]

Plastid ribosomal protein S5 is involved in photosynthesis, plant development, and cold stress tolerance in Arabidopsis — Plastid ribosomal protein S5 is involved in photosynthesis, plant development, and cold stress tolerance in Arabidopsis — Supplementary Data 

# Plastid ribosomal protein S5 is involved in photosynthesis, plant development, and cold stress tolerance in Arabidopsis

## Supplementary Data

Data files

- Supplementary Data - Supplementary Data
- Supplementary\_Figures\_S1\_S7\_Tables\_S1\_S2\_S4\_S6.pdf - Supplementary Data
