# Supplementary material for: Plastid ribosomal protein S5 is involved in photosynthesis, plant development, and cold stress tolerance in Arabidopsis
Source: J Exp Bot. 2016 Mar 22;67(9):2731–44. doi: 10.1093/jxb/erw106 (PMC4861020; doi:10.1093/jxb/erw106)
Supplement: Supplementary Data [file supp_erw106_Supplementary_Figures_S1_S7_Tables_S1_S2_S4_S6.pdf]

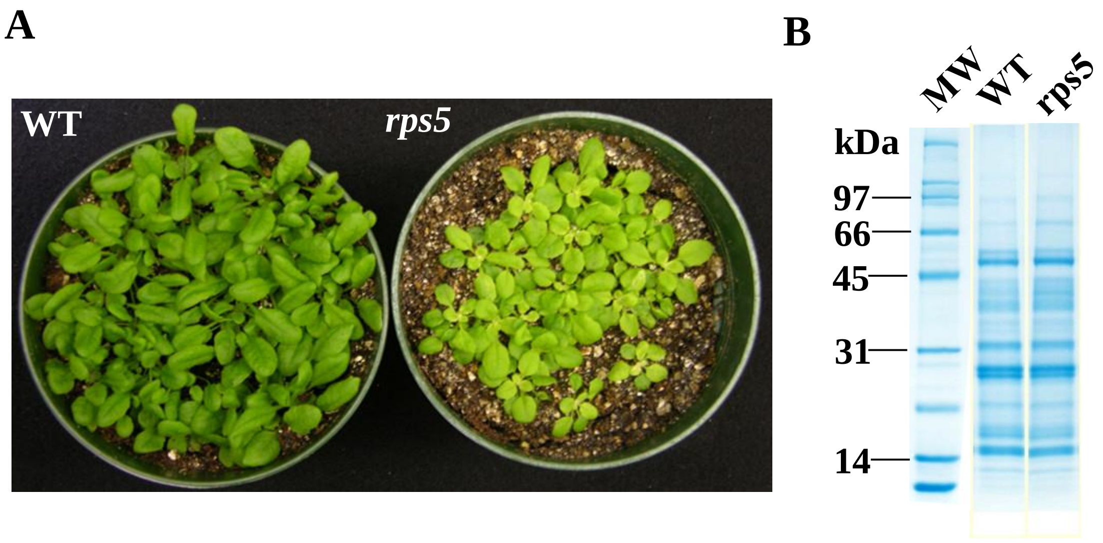

Supplementary Figure S1. The phenotype of 4-week-old wild-type and *rps5* mutant plants, and gel evaluation of proteins used for proteomic analysis. (A) Plants used for proteomic analysis. (B). Image of the extracted protein quality by 10-20% tris-glycine gel (B).

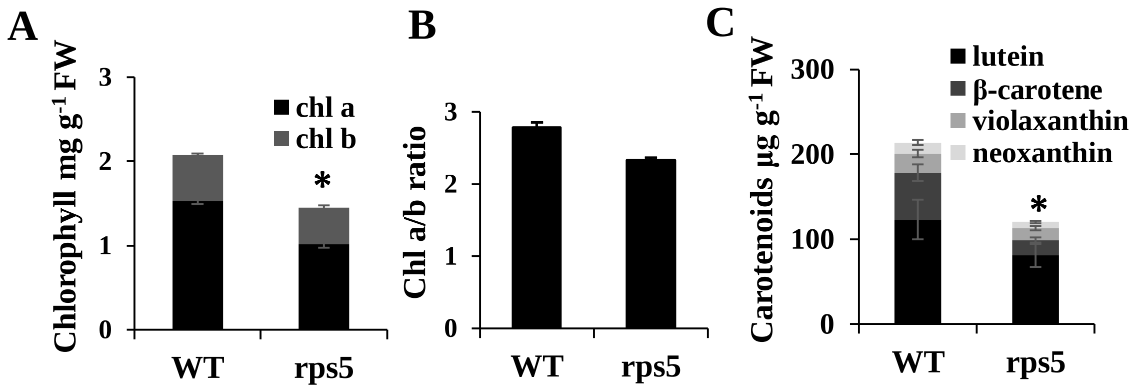

Supplementary Figure S2. Pigment content of wild-type and the *rps5* mutant. (A) Chlorophyll level. (B) Chlorophyll a/b ratio. (C) Carotenoid content and composition. Pigments were extracted from 4-week-old leaves of wild-type and *rps5* plants. Data represent means ± SD from three biological replicates. *indicates significant difference ($p < 0.05$).

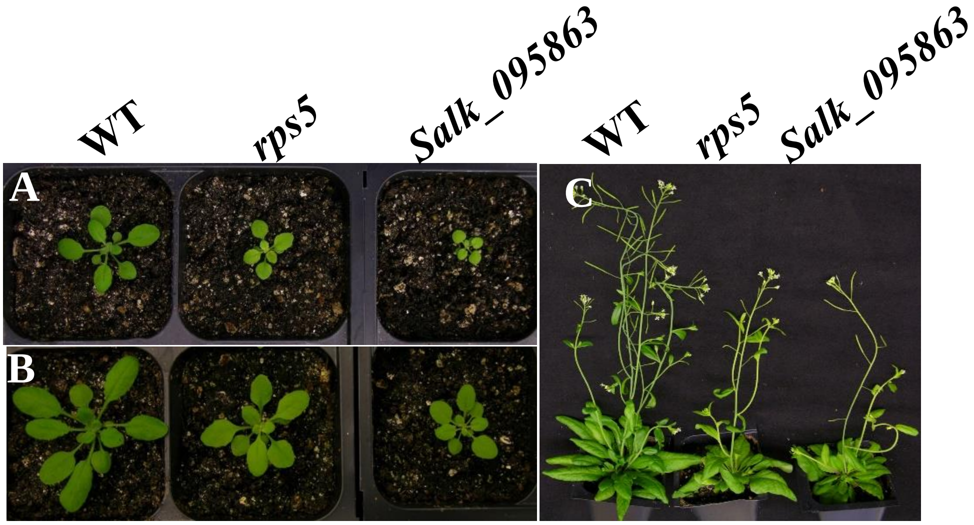

Supplementary Figure S3. The growth phenotype of wild-type, *rps5*, and *Salk_095863* mutants. (A) 3-week-old. (B) 4-week-old. (C) Mature plants.

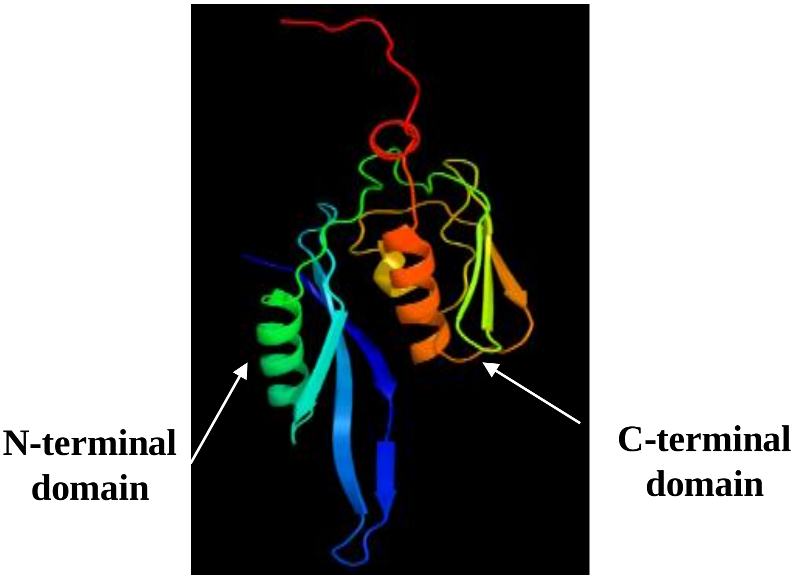

Supplementary Figure S4. RPS5 3D structure model. The 3D structure model for RPS5 protein was constructed using the Phyre2 server (http://www.sbg.bio.ic.ac.uk/phyre2/html/page.cgi?id=index)

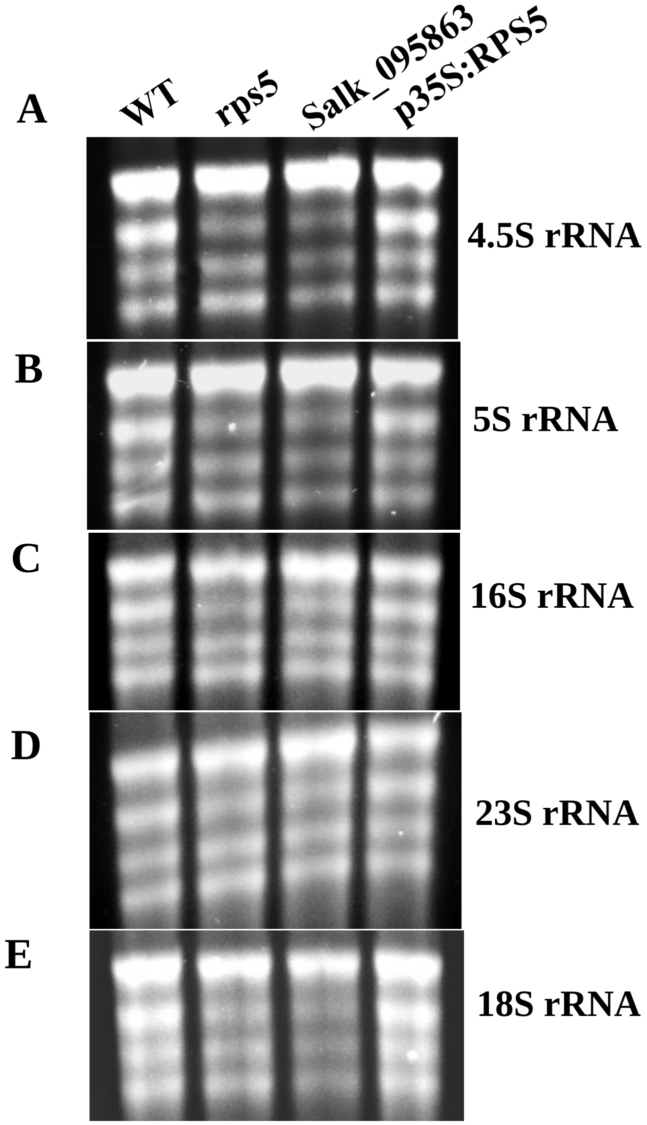

Supplementary Figure S5. RNA gel blotting loading control.

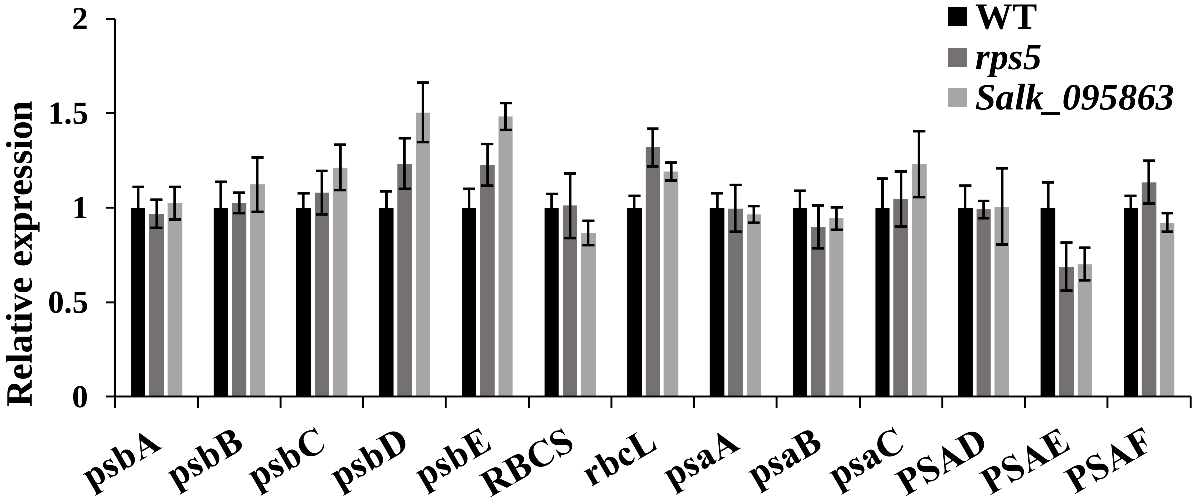

Supplementary Figure S6. Expression of photosynthesis-related genes in wild-type, *rps5*, and *Salk_095863*. qRT-PCR analysis of transcript levels of photosynthesis-related genes from 4-week-old plants. Values represent means ± SD from three biological replicates with three technical repeats.

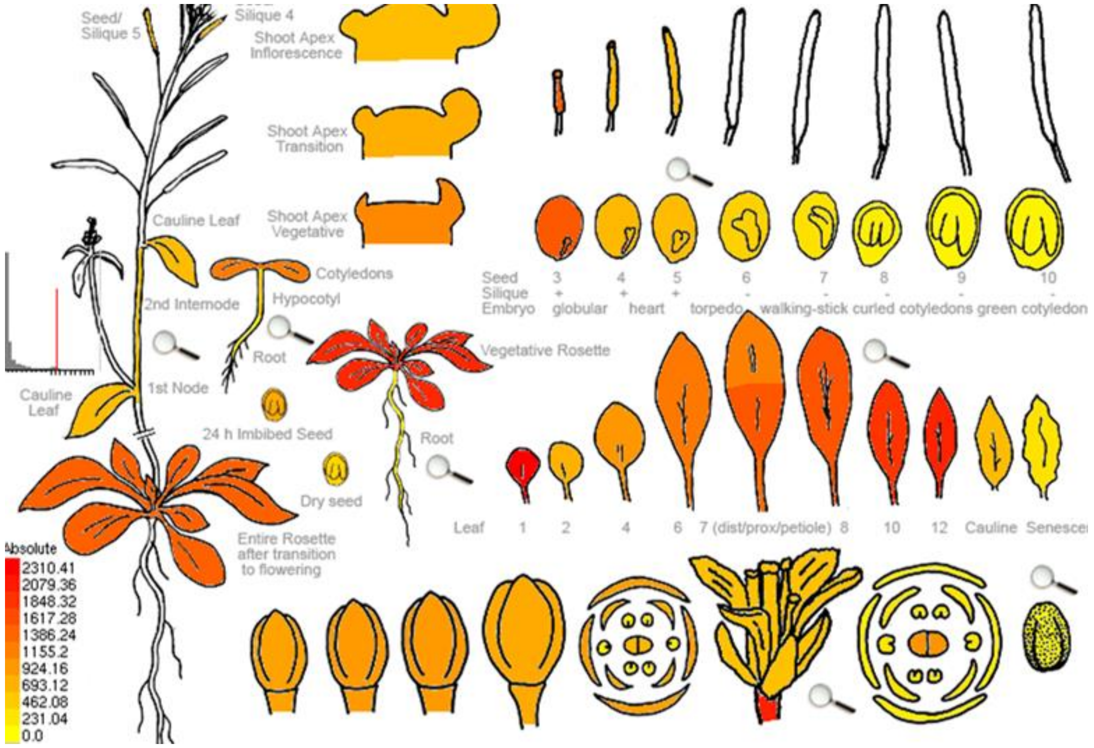

Supplementary Figure S7. Gene expression pattern of *RPS5* based on Arabidopsis e-FP Brower(http://bar.utoronto.ca/efp/cgi-bin/efpWeb.cgi).

Supplementary Table S1. Primers used in this study

Primer Name	Gene locus	Primer Sequence(5'-3')
	**Two co-segregation markers**	
13.30 Mb-F	AGATGAAGCTAAGGTGTTTGA	
13.30 Mb-R	GGGAGACAAAGAGCAATACCA	
13.32 Mb-F	TTGAAAGTTCCCGATTCTCG	
13.32 Mb-R	CGTAGTGAGAGCAACGACTTTC	
	**Cloning- complementation-p*35S::RPS5***	
RPS5-KpnI-cds-F	AT2G33800	GCAGGTACCATGGCGACAGCATCAGCTCTC
RPS5-PstI-cds-R	AT2G33800	GCACTGCAGTCACTTCCAGAGTTCTTCCATG
	**SALK T-DNA primer**	
LP	Salk_095863	AGCAGATTTCTGAACAGCAGC
RP	Salk_095863	AATTAACGTTGCTCGTTGGTG
	**Cloning- complementation-p*RPS5::RPS5***	
RPS5-KpnI-gDNA-F	AT2G33800	GCAGGTACCCCACGAAGCCAAAAGTGGATAT
RPS5-PstI-gDNA-R	AT2G33800	GCACTGCAGGCTATAGCATTCTGAAATAGAATC
	**Cloning-*RPS5/rps5-GFP***	
RPS5-GFP-XbaI-F	AT2G33800	GCTCTAGAATGGCGACAGCATCAGCTCT
RPS5-GFP-XmaI-R	AT2G33800	CGGACCCGGGCTTCCAGAGTTCTTCCATGG
	**RT-PCR/ qPCR**	
RPS5-F	AT2G33800	GTCGTAACCCTTTTGCCCTA
RPS5-R	AT2G33800	TGTGAATGTTGTCCGAAACTC
psaA-F	ATCG00350	GGGCGGTGAGTTAGTAGCAG
psaA-R	ATCG00350	TCACAAGGGAAACGAAAACC
psaB-F	ATCG00340	TATGCTCAAAACCCCGACTC
psaB-R	ATCG00340	TCCGATTCCAAAGTTCGTTC
psaC-F	ATCG01060	GAGCATGCCCTACAGACGTA
psaC-R	ATCG01060	TCGAGTTGTTTCATGCCATA
PSAD-2F	AT1G03130	AACAGGAGGAGCTGCGATAA
PSAD-2R	AT1G03130	TCCTGGATTCGCTTTCTCTG
PSAE-2F	AT2G20260	CCGCTAAGGCTAAACCTCCT
PSAE-2R	AT2G20260	CGTCCAATGCGTAGTTGTTG
PSAF-F	AT1G31330	GACGGTTTACCGCACTTGAT
PSAF-R	AT1G31330	CGGAAGATGATCCGACTAGC
psbA-F	ATCG00020	TCCGGTGCCATTATTCCTAC
psbA-R	ATCG00020	AGCAATCCAAGGACGCATAC

psbB-F	ATCG00680	GTGGAGGGTTTTGACCCTTT
psbB-R	ATCG00680	TCCAGCAACAACAAAAGCTG
psbD-F	ATCG00270	ACCGCTGCAGTTTCTACTCC
psbD-R	ATCG00270	TCGCAATTGAACAGATCGAG
psbC-F	ATCG00280	CTACCACGTGGAAACGCTCT
psbC-R	ATCG00280	CAAAATGGGCCACTTCAAAT
psbE-F	ATCG00580	TGTCTGGAAGCACAGGAGAA
psbE-R	ATCG00580	CGGCCTGTTATTAATGGAATG
rbcL-F	ATCG00490	TACCTGGTGTTCTGCCTGTG
rbcL-R	ATCG00490	GCAAGATCACGTCCCTCATT
RBCS1A-F	AT1G67090	ACCTTATCCGCAACAAGTGG
16S-F	ATCG00920	GGTCCTGAACTTCTTTTCCCAGAG
16S-R	ATCG00920	CTACAGACGCTTTACGCCCAAT
18S-F	AT2G01010	CTTTCGATGGTAGGATAGTGGC
18S-R	AT2G01010	GTCAGGATTGGGTAATTTGCG
23S-F	ATCG00950	TGAAACCGTAAGCTCCCAAG
23S-R	ATCG00950	ATTGCCCTATGAAGACTCGC

Supplementary Table S2. Blast analysis of *RPS5* in the Arabidopsis genome

Gene locus	Description	Score(bits)	E Value
AT2G33800.1	Ribosomal protein S5 family protein	293	1e-79
AT3G57490.1	Ribosomal protein S5 family protein	42	8e-04
AT2G41840.1	Ribosomal protein S5 family protein	42	8e-04
AT1G58684.1	Ribosomal protein S5 family protein	42	8e-04
AT1G58380.1	Ribosomal protein S5 family protein	42	8e-04
AT1G59359.1	Ribosomal protein S5 family protein	42	8e-04
AT1G58983.1	Ribosomal protein S5 family protein	42	8e-04
AT1G64880.1	Ribosomal protein S5 family protein	37	0.014

Supplementary Table S4. Proteins significantly down- and up-regulated in *rps5* compared with wild-type by iTRAQ proteomic analysis

Gene locus	Protein description	Ratio*
	**Down-regulated proteins in *rps5***	
ATCG00710.1	photosystem II reaction center protein H	0.36
AT2G30790.1	photosystem II subunit P-2	0.47
AT3G21055.1	photosystem II subunit T	0.51
AT1G06680.1	photosystem II subunit P-1	0.55
AT1G79040.1	photosystem II subunit R	0.55
AT1G51400.1	Photosystem II 5 kD protein	0.59
AT3G50820.1	photosystem II subunit O-2	0.6
ATCG00580.1	photosystem II reaction center protein E	0.6
AT1G76450.1	Photosystem II reaction center PsbP family protein	0.61
AT5G66570.1	PS II oxygen-evolving complex 1	0.61
AT4G05180.1	photosystem II subunit Q-2	0.64
AT4G21280.1	photosystem II subunit QA	0.65
AT1G03130.1	photosystem I subunit D-2	0.47
AT5G64040.1	photosystem I reaction center subunit N	0.61
AT4G12800.1	photosystem I subunit l	0.64
AT1G31330.1	photosystem I subunit F	0.74
AT4G28750.1	Photosystem I reaction centre subunit IV	0.48
AT4G02770.1	photosystem I subunit D-1	0.55
AT4G32260.1	Pigment defective 334	0.61
ATCG00120.1	ATP synthase subunit alpha	0.68
ATCG00480.1	ATP synthase subunit beta	0.73
AT2G47400.1	CP12 domain-containing protein 1	0.66
AT1G67090.1	Rubisco small subunit-4	0.68
AT4G34620.1	ribosomal protein S16B	0.53
AT2G33800.1	ribosomal protein S5	0.56
ATCG00900.1	ribosomal protein S7B	0.57
ATCG01120.1	ribosomal protein S15	0.58
AT1G79850.1	ribosomal protein S17	0.58
AT5G30510.1	ribosomal protein S1	0.69
ATCG00380.1	30S ribosomal protein S4	0.71
AT1G64510.1	ribosomal protein S6	0.6
ATCG00770.1	ribosomal protein S8	0.64
ATCG00650.1	ribosomal protein S18	0.65
AT3G15190.1	ribosomal protein S20	0.66
AT3G52150.1	PSRP-2 associates with 30S	0.66
AT1G74970.1	ribosomal protein S9	0.67
AT5G14320.1	ribosomal protein S13	0.71
AT5G56710.1	60S ribosomal protein L31	0.62
AT5G61170.1	40S ribosomal protein S19	0.73

AT3G49910.1	60S ribosomal protein L26	0.74
AT2G16360.1	40S ribosomal protein S25	0.743
AT3G53890.1	Ribosomal protein S21e	0.745
AT2G42540.1	cold-regulated 15A	0.29
AT2G42530.1	cold regulated 15B	0.36
AT1G76180.1	Dehydrin family protein	0.45
AT1G20450.1	Dehydrin family protein	0.46
AT5G52310.1	low-temperature-responsive protein 78	0.52
AT5G15970.1	cold-responsive 6.6 (COR6.6)	0.57
AT1G75040.1	pathogenesis-related gene 5	0.65
AT4G25050.1	acyl carrier protein 4	0.72
AT1G11840.1	glyoxalase I homolog	0.70
AT5G14910.1	Heavy metal transport	0.69
AT2G24940.1	membrane-associated progesterone binding protein 2	0.62
AT5G26667.1	uridylate kinase	0.69
AT3G57260.1	beta-1,3-glucanase 2	0.39
AT1G78370.1	glutathione S-transferase TAU 20	0.63
AT1G78380.1	glutathione S-transferase TAU 19	0.72
AT2G30860.1	glutathione S-transferase PHI 9	0.70
AT2G30870.1	glutathione S-transferase PHI 10	0.70
AT4G02520.1	glutathione S-transferase PHI 2	0.70
AT5G03630.1	monodehydroascorbate reductase	0.62
AT3G20390.1	Endoribonuclease L-PSP	0.74
AT2G24590.1	splicing factor, putative	0.70
AT2G06530.1	SNF7 family protein	0.71
AT2G21660.1	glycine-rich RNA-binding protein	0.64
AT5G27670.1	histone H2A 7	0.69
AT5G59870.1	histone H2A 6	0.73
AT1G66410.1	calmodulin 4	0.71
AT2G41100.1	Calcium-binding EF hand family protein	0.67
AT5G38480.1	general regulatory factor 3	0.69
AT1G16240.1	syntaxin of plants 51	0.72
AT3G58730.1	vacuolar ATP synthase subunit D	0.73
AT4G32470.1	Cytochrome bd ubiquinol oxidase	0.74
AT1G75950.1	S phase kinase-associated protein 1	0.73
AT1G22450.1	cytochrome C oxidase 6B	0.48
AT2G21870.1	ATP synthase 24 kDa subunit	0.67
AT3G52300.1	ATP synthase D chain	0.69
AT2G21530.1	SMAD/FHA domain-containing protein	0.68
AT4G20260.1	DREPP plasma membrane polypeptide family	0.73
AT5G23820.1	ML domain-containing protein	0.63
AT5G07020.1	proline-rich family protein	0.73
AT2G23670.1	homolog of Synechocystis YCF37	0.65
AT2G30930.1	unknown protein	0.73

AT4G01150.1	unknown protein	0.63
AT5G39570.1	unknown protein	0.53
AT1G67700.1	unknown protein	0.73
	**Up-regulated proteins in *rps5***	
AT5G04140.1	glutamate synthase 1	2.23
AT4G39040.1	RNA-binding CRS1 / YhbY domain protein	2.21
AT5G60600.1	4-hydroxy-3-methylbut-2-enyl diphosphate synthase	2.01
AT4G33010.1	glycine decarboxylase P-protein 1	2.01
AT1G56070.1	elongation factor 2	1.87
AT3G60750.1	Transketolase	1.82
AT2G42600.1	phosphoenolpyruvate carboxylase 2	1.77
AT1G16720.1	high chlorophyll fluorescence phenotype 173	1.76
AT2G26080.1	glycine decarboxylase P-protein 2	1.75
AT4G35090.1	catalase 2	1.73
AT1G01620.1	plasma membrane intrinsic protein 1C	1.72
AT3G19170.1	presequence protease 1	1.68
AT1G63770.1	Peptidase M1 family protein	1.67
AT4G26530.1	Aldolase superfamily protein	1.66
AT5G26742.1	DEAD box RNA helicase (RH3)	1.66
ATCG00340.1	psaB - subunit Ib	1.63
AT2G13360.1	alanine:glyoxylate aminotransferase	1.63
AT2G45960.1	plasma membrane intrinsic protein 1B	1.61
AT3G61220.1	NAD(P)-binding Rossmann-fold superfamily protein	1.60
AT4G24620.1	phosphoglucose isomerase 1	1.58
AT5G16970.1	alkenal reductase	1.58
AT1G06950.1	translocon at the inner envelope membrane of chloroplasts	1.57
AT1G79930.1	heat shock protein 91	1.57
AT1G70820.1	phosphoglucomutase-2	1.57
AT2G38230.1	pyridoxine biosynthesis 1.1	1.57
AT3G01500.1	carbonic anhydrase 1	1.56
AT1G76080.1	thioredoxin	1.55
AT1G69740.1	Aldolase superfamily protein	1.55
AT1G62750.1	elongation factor Tu-G	1.54
AT1G23310.1	glutamate:glyoxylate aminotransferase	1.54
AT3G48420.1	haloacid dehalogenase-like hydrolase-2	1.51
AT3G20820.1	Leucine-rich repeat family protein	1.49
AT5G14260.1	Rubisco methyltransferase family protein	1.49
AT2G39730.1	rubisco activase	1.48
AT5G67030.1	zeaxanthin epoxidase(ZEP)	1.47
AT1G42970.1	glyceraldehyde-3-phosphate dehydrogenase B	1.44
AT5G54770.1	thiazole biosynthetic enzyme	1.43
AT2G15620.1	nitrite reductase 1	1.43
AT2G04030.1	Chaperone protein htpG family protein	1.42
AT3G58140.1	Phe-tRNA synthetase	1.40

AT1G32060.1	phosphoribulokinase	1.39
AT2G39800.1	delta1-pyrroline-5-carboxylate synthase 1	1.39
AT4G37930.1	serine transhydroxymethyltransferase 1	1.39
AT5G35630.1	glutamine synthetase 2	1.39
AT1G68010.1	hydroxypyruvate reductase	1.38
AT2G43710.1	Plant stearoyl-acyl-carrier-protein desaturase family protein	1.38
AT5G26000.1	thioglucoside glucohydrolase 1	1.38
AT3G14067.1	Subtilase family protein	1.37
AT5G28840.1	GDP-D-mannose 3',5'-epimerase	1.37
ATCG00830.1	ribosomal protein L2A	1.37
AT5G55220.1	trigger factor type chaperone family protein	1.37
AT1G16080.1	unknown protein	1.36
AT1G48600.1	phosphoethanolamine N-methyltransferase 2	1.34
AT1G71695.1	Peroxidase superfamily protein	1.34
AT5G24300.1	Glycogen/starch synthases	1.33
AT1G11860.1	Glycine cleavage T-protein family	1.33
AT4G29060.1	elongation factor Ts family protein	1.33
AT1G48030.1	mitochondrial lipoamide dehydrogenase 1	1.33

* The protein ratio is rps5 / wild-type

Supplementary Table S5. Detailed information of functional classification of the down-regulated proteins in *rps5* proteome.

Gene locus	Protein description	Ratio*
	**PS pathways**	
AT1G06680.1	photosystem II subunit P-1	0.55
AT1G51400.1	Photosystem II 5 kD protein	0.59
AT1G76450.1	Photosystem II reaction center PsbP family protein	0.61
AT1G79040.1	photosystem II subunit R	0.55
AT2G30790.1	photosystem II subunit P-2	0.47
AT3G21055.1	photosystem II subunit T	0.51
AT3G50820.1	photosystem II subunit O-2	0.60
AT4G05180.1	photosystem II subunit Q-2	0.64
AT4G21280.1	photosystem II subunit QA	0.65
AT5G66570.1	PS II oxygen-evolving complex 1	0.61
ATCG00580.1	photosystem II reaction center protein E	0.60
ATCG00710.1	photosystem II reaction center protein H	0.36
AT1G03130.1	photosystem I subunit D-2	0.47
AT1G31330.1	photosystem I subunit F	0.74
AT4G02770.1	photosystem I subunit D-1	0.55
AT4G12800.1	photosystem I subunit l	0.64
AT4G28750.1	Photosystem I reaction centre subunit IV / PsaE protein	0.48
AT5G64040.1	photosystem I reaction center subunit PSI-N,	0.61
AT4G32260.1	ATPase, F0 complex, subunit B/B'	0.61
ATCG00120.1	ATP synthase subunit alpha	0.68
ATCG00480.1	ATP synthase subunit beta	0.73
AT2G47400.1	CP12 domain-containing protein 1	0.66
AT1G67090.1	ribulose bisphosphate carboxylase small chain 1A	0.68
	**Protein**	
AT4G34620.1	ribosomal protein S16B	0.53
AT2G33800.1	ribosomal protein S5	0.56
ATCG00900.1	ribosomal protein S7B	0.57
ATCG01120.1	ribosomal protein S15	0.58
AT1G79850.1	ribosomal protein S17	0.58
AT5G30510.1	ribosomal protein S1	0.69
ATCG00380.1	30S ribosomal protein S4	0.71
AT1G64510.1	ribosomal protein S6	0.6
ATCG00770.1	ribosomal protein S8	0.64
ATCG00650.1	ribosomal protein S18	0.65
AT3G15190.1	ribosomal protein S20	0.66
AT3G52150.1	PSRP-2 associates with 30S	0.66
AT1G74970.1	ribosomal protein S9	0.67
AT5G14320.1	ribosomal protein S13	0.71
AT5G56710.1	60S ribosomal protein L31	0.62

AT5G61170.1	40S ribosomal protein S19	0.73
AT3G49910.1	60S ribosomal protein L26	0.74
AT2G16360.1	40S ribosomal protein S25	0.74
AT3G53890.1	Ribosomal protein S21e	0.74
AT1G75950.1	S phase kinase-associated protein 1	0.72
	**Stress**	
AT2G42540.1	cold-regulated 15a	0.29
AT2G42530.1	cold regulated 15b	0.36
AT5G52310.1	cold-regulated 78	0.52
AT5G15970.1	cold-regulated 6.6	0.57
AT1G76180.1	Dehydrin family protein	0.45
AT1G20450.1	Dehydrin family protein	0.46
AT1G75040.1	pathogenesis-related gene 5	0.65
	**misc**	
AT4G32470.1	Cytochrome bd ubiquinol oxidase	0.74
AT1G22450.1	cytochrome C oxidase 6B	0.48
AT2G21870.1	copper ion binding	0.67
AT3G52300.1	ATP synthase D chain	0.69
	**RNA**	
AT3G20390.1	endoribonuclease L-PSP family protein	0.74
AT2G24590.1	splicing factor, putative	0.70
AT2G06530.1	SNF7 family protein	0.71
AT2G21660.1	glycine-rich RNA-binding protein	0.64
	**Signalling**	
AT1G66410.1	calmodulin 4	0.71
AT2G41100.1	Calcium-binding EF hand family protein	0.67
AT5G38480.1	general regulatory factor 3	0.69
	**DNA**	
AT5G27670.1	histone H2A 7	0.69
AT5G59870.1	histone H2A 6	0.73
	**Redox regulation**	
AT5G03630.1	monodehydroascorbate reductase	0.62
AT2G24940.1	cytochrome b5 domain-containing	0.62
	**Transport**	
AT3G58730.1	vacuolar ATP synthase subunit D	0.73
	**Cell**	
AT1G16240.1	syntaxin of plants 51	0.72
	**Nucleotide metabolism**	
AT5G26667.1	uridylate kinase	0.69
	**Lipid metabolism**	
AT4G25050.1	acyl carrier protein 4	0.72
	**Amino acid metabolism**	

AT1G11840.1	glyoxalase I homolog	0.70
	**Metal handling**	
AT5G14910.1	detoxification superfamily protein	0.69
	**Not assigned**	
AT1G67700.1	unknown protein	0.73
AT2G21530.1	unknown protein	0.68
AT4G20260.1	DREPP plasma membrane polypeptide family	0.73
AT5G23820.1	ML domain-containing protein	0.63
AT5G07020.1	proline-rich protein family	0.73
AT2G23670.1	unknown protein	0.65
AT2G30930.1	unknown protein	0.73
AT4G01150.1	unknown protein	0.63
AT5G39570.1	unknown protein	0.53

Supplementary Table S6. Detailed information of functional classification of up-regulated proteins in *rps5* proteome.

Gene locus	Protein description	Ratio*
	**PS pathways**	
ATCG00340.1	psaB - subunit Ib	1.63
AT1G23310.1	glutamate:glyoxylate aminotransferase	1.54
AT2G13360.1	alanine:glyoxylate aminotransferase	1.63
AT1G68010.1	hydroxypyruvate reductase	1.38
AT1G32060.1	phosphoribulokinase	1.39
AT2G39730.1	rubisco activase	1.48
AT1G42970.1	glyceraldehyde-3-phosphate dehydrogenase B	1.44
AT3G60750.1	Transketolase	1.82
AT1G06680.1	photosystem II subunit P-1	0.55
	**Protein**	
AT3G58140.1	Phe-tRNA synthetase	1.40
ATCG00830.1	ribosomal protein L2	1.37
AT1G56070.1	elongation factor 2, EF-2	1.87
AT1G62750.1	elongation factor Tu-G (EF-G)	1.54
AT4G29060.1	elongation factor Ts family protein	1.33
AT1G16720.1	high chlorophyll fluorescence phenotype 173	1.76
AT1G06950.1	Tic110	1.57
AT1G63770.1	Peptidase M1 family protein	1.67
AT3G14067.1	Subtilase family protein	1.37
AT3G19170.1	presequence protease 1	1.68
AT2G04030.1	Chaperone protein htpG family protein	1.42
AT5G55220.1	trigger factor type chaperone family protein	1.37
	**Glycolysis**	
AT2G42600.1	phosphoenolpyruvate carboxylase 2	1.77
AT1G70820.1	phosphoglucomutase, putative	1.57
AT4G24620.1	phosphoglucose isomerase 1	1.58
AT4G26530.1	Aldolase superfamily protein	1.66
	**Amino acid metabolism**	
AT2G39800.1	delta1-pyrroline-5-carboxylate synthase 1	1.39
AT1G11860.1	Glycine cleavage T-protein family	1.33
AT2G26080.1	glycine decarboxylase P-protein 2	1.75
AT4G33010.1	glycine decarboxylase P-protein 1	2.01
	**RNA**	
AT5G14260.1	Rubisco methyltransferase family protein	1.49
AT4G39040.1	RNA-binding CRS1	2.21
AT5G26742.1	DEAD box RNA helicase (RH3)	1.66
	**Redox regulation**	
AT1G76080.1	thioredoxin (CDSP32)	1.55

AT5G28840.1	GDP-mannose 3,5-epimerase	1.37
AT4G35090.1	catalase 2 (CAT2)	1.73
**N-metabolism**		
AT2G15620.1	nitrite reductase 1	1.43
AT5G04140.1	glutamate synthase 1	2.23
AT5G35630.1	glutamine synthetase 2	1.39
**Secondary metabolism**		
AT5G60600.1	4-hydroxy-3-methylbut-2-enyl diphosphate synthase	2.01
AT5G67030.1	zeaxanthin epoxidase (ZEP) (ABA1)	1.47
AT5G26000.1	thioglucoside glucohydrolase 1	1.38
**TCA**		
AT1G48030.1	mitochondrial lipoamide dehydrogenase 1	1.33
AT3G01500.1	carbonic anhydrase 1	1.56
**Transport**		
AT1G01620.1	plasma membrane intrinsic protein 1C	1.72
AT2G45960.1	plasma membrane intrinsic protein 1B	1.61
**misc**		
AT1G71695.1	Peroxidase superfamily protein	1.34
AT5G16970.1	alkenal reductase	1.58
**Vitamine metabolism**		
AT5G54770.1	thiazole biosynthetic enzyme	1.43
AT2G38230.1	pyridoxine biosynthesis 1.1	1.57
**Lipid metabolism**		
AT2G43710.1	stearoyl-ACP desaturase	1.38
AT1G48600.1	phosphoethanolamine N-methyltransferase 2	1.34
**Stress**		
AT3G61220.1	NAD(P)-binding Rossmann-fold superfamily protein	1.60
AT1G79930.1	Heat shock hsp70 protein	1.57
**C1-metabolism**		
AT4G37930.1	serine transhydroxymethyltransferase 1	1.39
**Tetrapyrrole synthesis**		
AT1G69740.1	Aldolase superfamily protein	1.55
**Major CHO metabolism**		
AT5G24300.1	Glycogen/starch synthases	1.34
**Not assigned**		
AT3G20820.1	leucine-rich repeat family protein	1.49
AT3G48420.1	haloacid dehalogenase-like hydrolase-2	1.51
AT1G16080.1	unknown protein	1.36
